# IL-10 and integrin signaling pathways are associated with head and neck cancer progression

**DOI:** 10.1186/s12864-015-2359-6

**Published:** 2016-01-08

**Authors:** Sophia Bornstein, Mark Schmidt, Gabrielle Choonoo, Trevor Levin, Joe Gray, Charles R. Thomas, Melissa Wong, Shannon McWeeney

**Affiliations:** Department of Radiation Medicine, Oregon Health & Science University, 3181 SW Sam Jackson Park Road, Portland, OR 97239 USA; Department of Cell, Developmental & Cancer Biology, Oregon Health & Science University 3, 3181 SW Sam Jackson Park Road, Portland, OR 97239 USA; OHSU Knight Cancer Institute, Oregon Health & Science University, 3181 SW Sam Jackson Park Road, Portland, OR 97239 USA; Department of Biomedical Engineering, Oregon Health & Science University, 3181 SW Sam Jackson Park Road, Portland, OR 97239 USA; Division of Bioinformatics and Computational Biology, Department of Medical Informatics & Clinical Epidemiology, Oregon Health & Science University, 3181 SW Sam Jackson Park Road, Portland, OR 97239 USA; Division of Biostatistics, Department of Public Health and Preventive Medicine, Oregon Health & Science University, 3181 SW Sam Jackson Park Road, Portland, OR 97239 USA

**Keywords:** HNSCC, CTCF, Head and neck cancer, IL-10, Integrin, RNA-seq, TCGA, Mutation, Signature

## Abstract

**Background:**

Head and neck cancer is morbid with a poor prognosis that has not significantly improved in the past several decades. The purpose of this study was to identify biological pathways underlying progressive head and neck cancer to inform prognostic and adjuvant strategies. We identified 235 head and neck cancer patients in The Cancer Genome Atlas (TCGA) with sufficient clinical annotation regarding therapeutic treatment and disease progression to identify progressors and non-progressors. We compared primary tumor gene expression and mutational status between these two groups.

**Results:**

105 genes were differentially expressed between progressors and nonprogressors (FDR < 0.05). Pathway analyses revealed deregulation (FDR < 0.05) of multiple pathways related to integrin signaling as well as IL-10 signaling. A number of genes were uniquely mutated in the progressor cohort including increased frequency of truncating mutations in CTCF (*P* = 0.007). An 11-gene signature derived from a combination of unique mutations and differential expression was identified (*PAGE4, SMTNL1, VTN, CA5A, C1orf43, KRTAP19-1, LEP, HRH4, PAGE5, SEZ6L, CREB3*). This signature was associated with decreased overall survival (Logrank Test; *P* = 0.03443). Cox modeling of both key clinical features and the signature was significant (*P* = 0.032) with the greatest prognostic improvement seen in the model based on nodal extracapsular spread and alcohol use alone (*P* = 0.004).

**Conclusions:**

Molecular analyses of head and neck cancer tumors that progressed despite treatment, identified IL-10 and integrin pathways to be strongly associated with cancer progression. In addition, we identified an 11-gene signature with implications for patient prognostication. Mutational analysis highlighted a potential role for *CTCF*, a crucial regulator of long-range chromatin interactions, in head and neck cancer progression.

**Electronic supplementary material:**

The online version of this article (doi:10.1186/s12864-015-2359-6) contains supplementary material, which is available to authorized users.

## Background

The 5-year survival rate for primary locally advanced head and neck squamous cell carcinoma (HNSCC) is approximately 50 % [1], however recurrent disease carries a dismal prognosis of 10.1 months with first line chemotherapy [2]. HNSCC recurs ~30 % of the time, most often within the first 1–2 years of definitive treatment. Pathways associated with progression have been identified using array-based gene expression analysis; however these studies are limited by the lack of rigor using older analysis techniques and normalization techniques, and heterogeneously treated patients. Identification of specific pathways linked to progression after radiation has the promise of informing targeted strategies to improve the prognosis of head and neck cancer.

The Cancer Genome Atlas (TCGA) is a joint effort of the National Cancer Institute and the National Human Genome Research Institute and has revolutionized the ability of investigators to ask prognostic questions about tumor biology previously limited by suboptimal sample sizes, non-standard sample normalization, and outdated techniques. HNSCC lends itself to this study, as there are diverse epidemiologic risk factors (e.g. HPV vs. tobacco), anatomic subsites (e.g. larynx vs. oral cavity), and issues with heterogeneity even within subpopulations of tumor cells within a given patient. To this end, TCGA has generated whole exome sequencing, SNP array, DNA methylation, RNA-Seq, and miRNA-Seq data for a large collaborative cohort of HNSCC patients. Their results have recently been published highlighting distinct subgroups within newly diagnosed HNSCC patients e.g. different mutational profiles between HPV-driven and tobacco-related tumors [3]. TCGA annotated data on these patients provides an unprecedented opportunity to determine which molecular pathways are most associated with disease progression and survival, in order to gain insight into potentially targetable biology. We sought to determine what molecular alterations were unique among HNSCC progressors in TCGA to help inform future patient stratification and adjuvant treatment.

## Results and discussion

### Patient demographics

We annotated 235 patients in TCGA with a median follow up time of 530.5 days and determined that 29 % of them had progressed (representing the “progressor” cohort, Table 1, Additional file 1: Table S1 and S2). The mean age was 62 years old, 74 % of patients were male, 90 % where white, and 77 % of patients smoked. Primary sites included the oral cavity (57 %), oropharynx (18 %), and larynx (25 %). Unfortunately, 71 % of patients did not have known HPV status (p16 staining or ISH). Stage distribution included 41 % stage I-III and 57 % stage IV, with 17 % having close or positive margins and 20 % with gross or microscopic nodal extracapsular extension. At the time of last follow up, 30.5 % of patients were deceased with a median of 456 days to death. Radiation treatment was part of initial treatment in 70 % of patients and was used as a single modality in 26 % of patients. Chemotherapy was used in 43 % of patients but only in 2 % as a single modality. Among the progressor cohort, primary sites similarly included oral cavity (56 %), oropharynx (19 %), and larynx (25 %). Stage distribution was 36.5 % stage I-III and 62 % stage IV, with 29.5 % having close or positive margins and 36 % with gross or microscopic nodal extracapsular extension. Radiation treatment was used in 88 % of these patients. Median follow up was 411 days and 62 % of patients were deceased at a median of 475.5 days to death. The overall demographics reflect what we would expect clinically, and we used this sample set to make comparisons between progressors and nonprogressors.

**Table 1:** Demographics for TCGA HNSCC patients analyzed in this study (progressors and nonprogressors)

	Patients (*n* = 235)
Age (mean)	20–90 (62)
Gender (M/F)	173(74 %)/62(26 %)
Race (W/B/A/AI/NA)^a^	212(90 %)/12(4.5 %)/4(2 %)/2(1 %)/5(2.5 %)
Smoke (Y/N/NA)	181(77 %)/52(22 %)/2(1 %)
Alcohol (Y/N/NA)	169(71.5 %)/61(26 %)/5(2.5 %)
HPV p16 or ISH (+/−/NA)	19(8 %)/49(21 %)/167(71 %)
Site (OC/OPX/L)^b^	135(57 %)/41(18 %)/59(25 %)
T Stage (T1-T2/T3/T4/TX/NA)	80(34 %)/64(27 %)/87(37 %)/3(1.5 %)/1(0.5 %)
N Stage (N0/N+/NA)	107(45.5 %)/127(54 %)/1(0.5 %)
Tumor Stage (I-III/IV/NA)	96(41 %)/135(57 %)/4(2 %)
Margin Status (+/−/Close/NA)	17(7 %)/158(67 %)/22(10 %)/38(16 %)
Nodal Extracapsular Spread (GE/ME/NE/NA)^c^	13(6 %)/34(14 %)/114(49 %)/74(31 %)
Curated Therapy: Therapy (C/R/CR/CRTM/CRTMV/NA)^d^	5(2 %)/60(26 %)/95(40 %)/1(0.5 %)/1(0.5 %)/73(31 %)
Radiation data: Radiation Dose cGy (mean)	9–7380 (4720)
Follow-up data: Follow-up Days (median)	45–4241 (530.5)
Follow-up data: Mortality (Living/Deceased)	164(69.5 %)/71(30.5 %)
Follow-up data: Days to Death (median)	23–5152 (456)
Follow-up data: Days to New Tumor (median)	50–1859 (339)
Radiation Treatment (Y/N)	165(70 %)/70(30 %)
Progression (Y/N)	68(29 %)/167(71 %)

^a^ *W* white, *B* black, *A* asian, *AI* American Indian

^b^ *OC* oral cavity, *OPX* oropharynx, *L* larynx

^c^ *GE* gross extension, *ME* microscopic extension, *NE* no extranodal Extension

^d^ *C* chemotherapy only, *R* radiation only, *CR* chemotherapy & radiation, *CRTM* chemotherapy, radiation & targeted molecular therapy, *CRTMV* chemotherapy, radiation, targeted molecular therapy & vaccine

Gray Shaded rows indicate significant differences between progressors and nonprogressors (*P* < 0.05)

### Differentially expressed genes

**Fig. 1:**
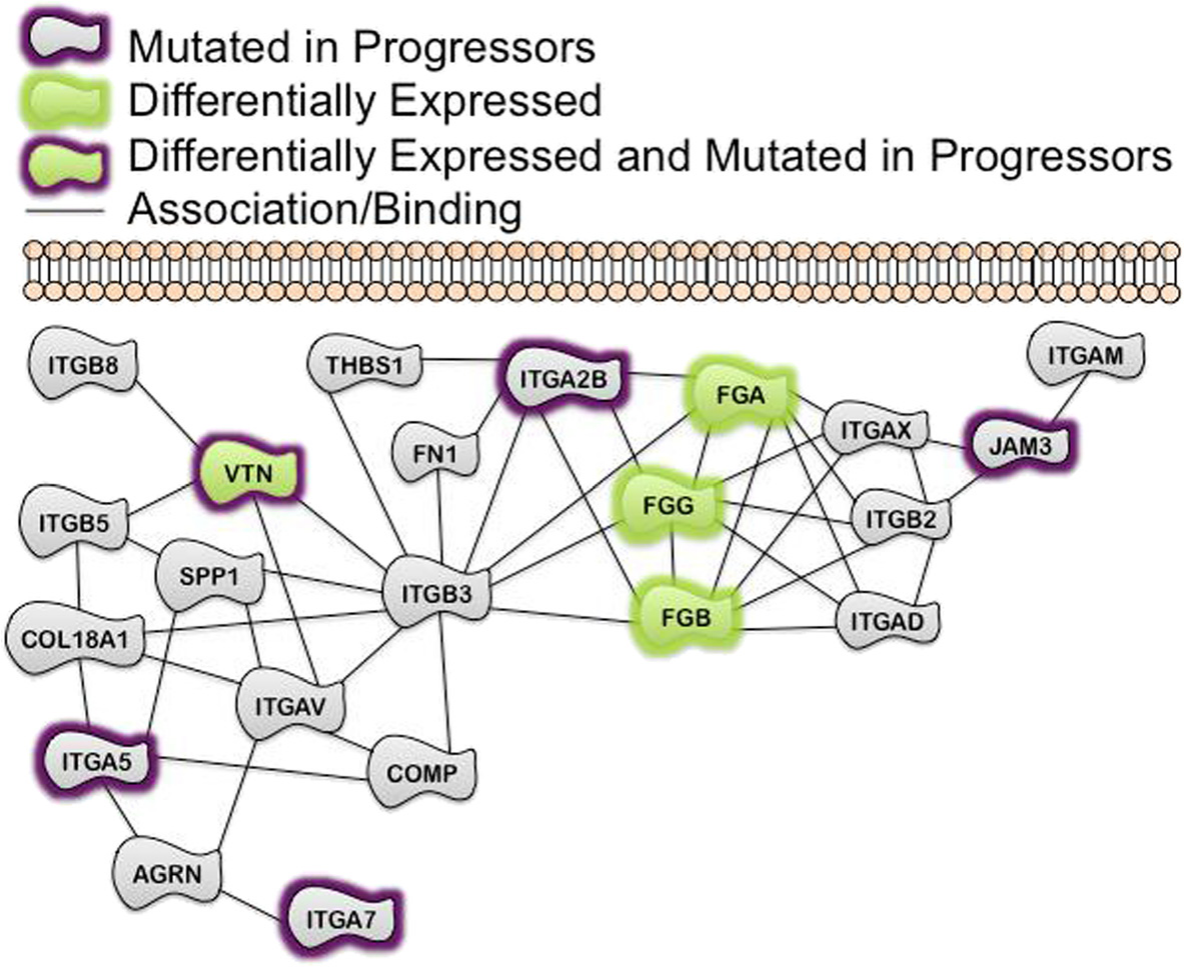
Examination of Differentially expressed (Progressors vs NonProgressors) and mutated genes in TCGA HNSCC subjects in the context of a stylized pathway representation of the Reactome Integrin Cell Surface Interactions Pathway, which was significantly enriched for the putative DE candidate genes (False Discovery Rate Adjusted *P*-value = 0.00424). Note that three of these genes are also CTCF binding sites: VTN, FGG, and FGB

**Fig. 2:**
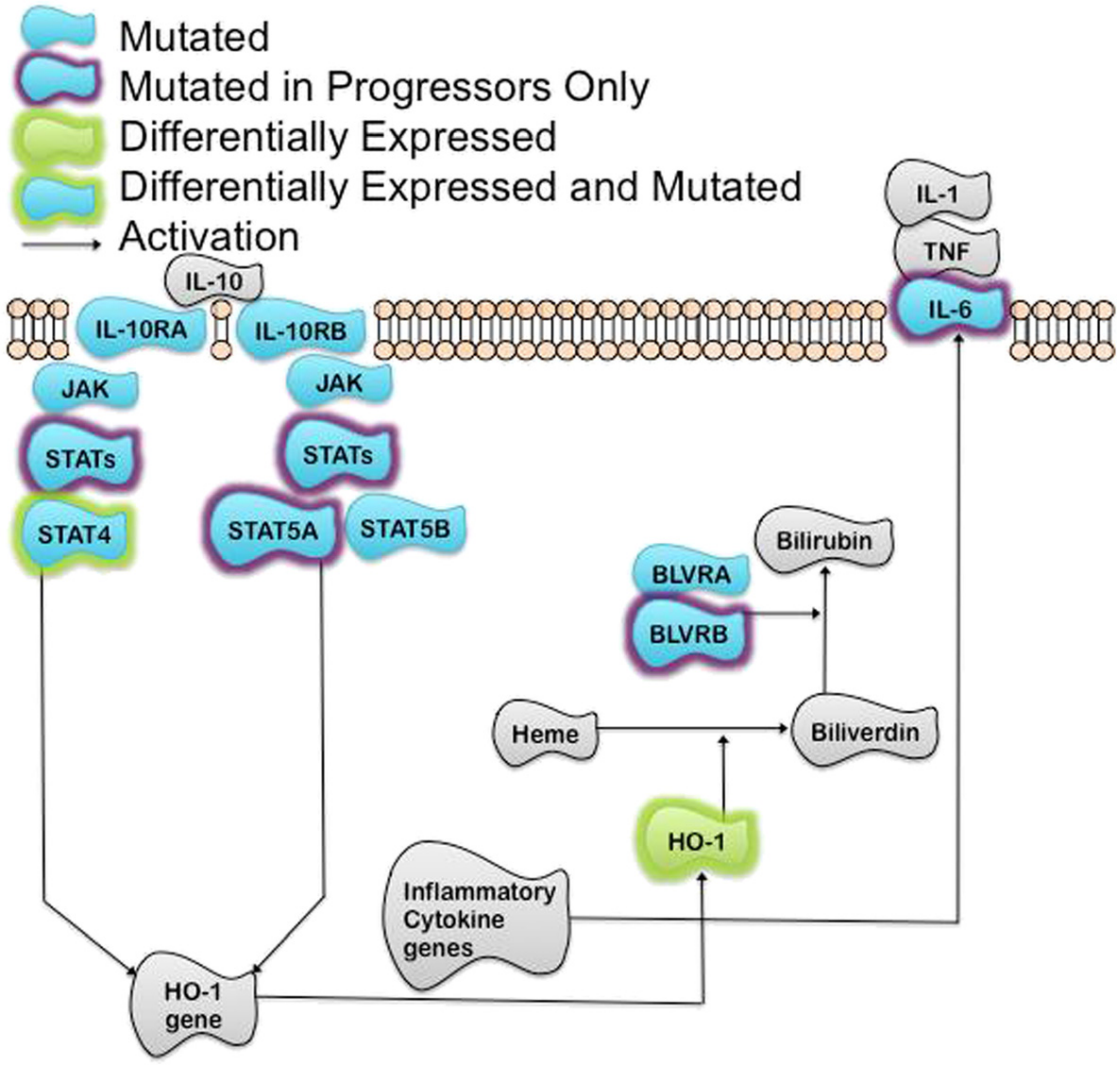
The IL-10 Anti-inflammatory Signaling Pathway is enriched for putative differentially expressed genes (Progressors vs NonProgressors Enrichment False Discovery Rate adjusted *P*-value = 0.0287) Figure is a stylized representation of the Biocarta pathway. Note the following synonyms: JAK: JAK1, HO-1: HMOX1, IL-1: IL1A. Also note that STAT4 is also a CTCF binding site

For the primary comparison of progressors versus nonprogressors, 105 differentially expressed candidate genes were identified (Additional file 2: Table S3). To provide biological context, we examined if curated pathway models were enriched for any of the genes in the candidate gene list. There were a striking number of Integrin-related pathways (from both PID and Reactome) that were significantly enriched for several of the differentially expressed candidate genes (Additional file 3: Table S4, and stylized representation in Fig. 1). In addition, the Biocarta Fibrinolysis pathway, IL-10 Anti-inflammatory Signaling Pathway (Fig. 2), as well as the KEGG Complement and Coagulation Cascades were among other pathways identified as significantly enriched (Additional file 3: Table S4). We then were interested in determining whether these pathways were reflected in progressors assigned to radiation treatment, as this is the primary adjuvant treatment modality in HNSCC.

### Radiation treatment assignment

We examined differential expression in progressors and nonprogressors assigned to radiation treatment to determine the extent of overlap in enriched pathways between this subset and the entire group. There were 60 progressors assigned radiation treatment and 105 nonprogressors. From the 492 differentially expressed genes (based on FDR < 0.05, data not shown), the overlap of differentially expressed genes from the 105 list with the 492 list was 15.2 %., With respect to shared pathways, we identified that the KEGG Complement and Coagulation Cascades pathway was also enriched in the progressors assigned to radiation treatment (Additional file 3: Table S4 and S5). Other pathways enriched in progressors assigned to radiation included MAPK signaling (KEGG), Cell Adhesion (KEGG), FGFR Ligand Binding and Activation, GPCR ligand binding, PI-3 K Cascades, and Cell-Cell Junction Organization (all Reactome) (Additional file 3: Table S5). Of note, in this cohort as well, interactions with the extracellular environment appeared to be important for progression. While this was an interesting finding, given our modest sample size after stratification by treatment assignment, we carried out the rest of our analyses with the entire cohort. We next moved to look at somatic mutations in the entire cohort, comparing progressors and nonprogressors.

### Somatic mutations

We examined the somatic mutations in progressors and nonprogressors to examine both shared and “unique” mutations at both the variant and gene level (Additional file 4: Table S6). We note that “unique” is defined as a mutation only being seen in one group and could reflect sampling issues. When we examined the ratio of variant to gene level mutation for the unique mutations, the ratio was higher for progressors than for nonprogressors (7.97 to 4.99). This is in contrast to the larger range of mutations seen in nonprogressors as well a higher median number of mutations (Additional file 4: Table S6). This suggests that even though there are more mutations seen in the nonprogressors (also a larger sample size), there appears to be higher variability relative to the number of genes mutated in progressors compared to nonprogressors. When pathways were examined for enrichment of somatic mutations (where the frequency of mutations between progressors and nonprogressors was >5 %), pre-NOTCH Transcription and Translation (Reactome), as well as ECM Receptor Interaction (KEGG) were among those identified (Table 2), again highlighting the significance of microenvironment interactions.

**Table 2:** Pathways enriched for genes with increased frequency of mutations in the Progressor cohort (Differential > 5 %) compared to NonProgressors

Pathway (Source)	FDR adjusted *P*-value	Gene Members
Pre-NOTCH Transcription and Translation (Reactome)	0.00853	CREBBP, NOTCH2, TP53*
Ion transport by P-type ATPases (Reactome)	0.00853	ATP10B, ATP2C1, ATP8B4**
ECM Receptor Interaction (KEGG)	0.0432	RELN, LAMA2, **ITGA7**
Glycosaminoglycan metabolism (Reactome)	0.049	CHSY3, CSGALNACT1, **B3GNT7**

Bold indicates genes found mutated only in the Progressor cohort

*FDR* false discovery rate

*Same genes are also enriched in Pre-NOTCH Expression and Processing (Reactome, FDR adjusted *P*-value = 0.0125)

**Same genes are also enriched in Ion Channel Transport (Reactome, FDR adjusted *P*-value =0.0183)

**Fig. 3:**
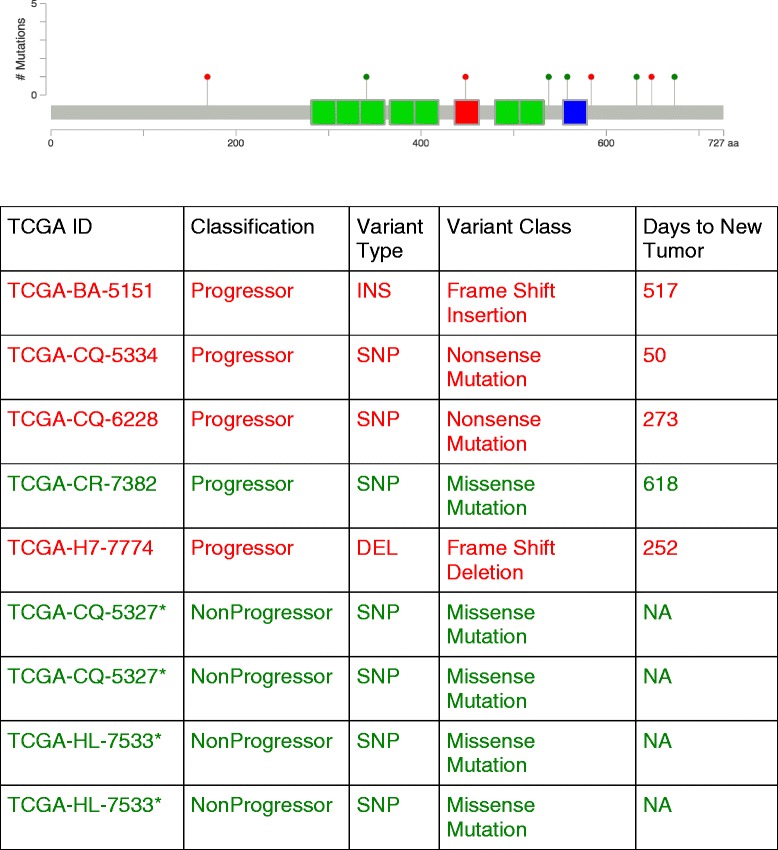
Lollipop plot highlighting mutations on a linear protein and its domains to examine somatic mutations within the CTCF gene in TCGA HNSCC Annotated Progressors and NonProgressors. Green indicates Missense mutations and Red indicates a truncating mutation. Note 2 silent mutations (one in Progressor TCGA-CQ-5334 who also had a frame shift insertion and one in a NonProgressor (TCGA-CN-A63T) are not shown. Plot was generated by the MutationMapper visualization tool

Among the somatic mutations, the distribution for only the truncating mutations was examined (Additional file 4: Table S6). Again a larger ratio of variants to gene level mutations was seen in progressors compared to nonprogressors (1.68 to 1.52). We then examined which genes had a significant increase in truncating mutations in progressors compared to nonprogressors. We filtered the list of genes examined to those genes significant by Mutsig annotation (i.e. gene mutated above what would be expected for background mutational processes). For the gene CCCTC-binding factor (*CTCF*), zinc finger chromatin-binding factor and transcriptional regulator, there was a significant occurrence of truncating mutations (*P* = 0.007) in progressors, as well as an increased overall frequency of mutations in the progressors compared to the nonprogressors (*P* = 0.02). All of the truncating mutations were seen in progresssors (Fig. 3). *CTCF* was ranked 21st of all mutations in the HNSCC complete TCGA cohort based on MutSig2CV analysis (FDR adjusted *P*-value = 0.04). We then examined the differentially expressed candidate genes for those with *CTCF* binding sites (Additional file 2: Table S3). When only these genes were utilized in the pathway enrichment analysis, Integrin-related pathways were again identified from both PID and Reactome (Table 3). After curating the differential expression and mutational analysis for progressors and nonprogressors, we chose to focus on Integrin and IL-10 *pathways, given these pathways were clearly enriched in the entire progressor cohort.*

**Table 3:** Pathways enriched for DE genes (Progressor vs NonProgressor) with CTCF Binding Sites

Pathway (Source)	FDR adjusted *P*-value	Gene Members
Beta3 integrin Cell Surface Interactions (PID)	0.00524	FGG, FGB, **VTN** ^a^
P130Cas linkage to MAPK signaling for Integrins (Reactome)	0.0107	FGG, FGB, **VTN** ^a^
Response to Elevated Platelet Cytosolic CA2+ (Reactome)	0.0107	FGG, FGB, ALB
Fibrinolysis Pathway (Biocarta)	0.0107	FGG, FGB

Bold indicates genes found mutated only in the Progressor cohort

*FDR* false discovery rate

^a^Same genes also enriched in Urokinase-type Plasminogen activator (uPA) and UPAR-mediated signaling (PID, FDR adjusted *P*-value = 0.00524); Beta1 integrin Cell Surface Interactions (PID, FDR adjusted *P*-value = 0.0107); Integrin Cell Surface Interactions (Reactome, FDR adjusted *P*-value = 0.0107)

### Integrin signaling deregulation in HNSCC progressors

As mentioned above, the Reactome Integrin Cell Surface Interaction pathway was significantly enriched for the putative differentially expressed candidate genes (FDR adjusted *P*-value = 0.00424, Fig. 1). In addition, the frequency of uniquely mutated genes in progressors ranged from 1.4 to 5.8 %, with 26.5 % of the progressors having at least one gene mutated in the pathway (of those, the median was 4.8 % and the range was 4.8–9.5 %). For the progressors, 45.6 % had overexpression of at least one gene in this pathway representation (of those, the median was 9 % of the pathway overexpressed with a range of 4.5 %–27.3 %). When evaluating combined mutation and overexpression, 61.8 % of the progressors had at least one gene aberrant in this pathway. In addition to this specific Reactome pathway, four other Reactome pathways relating to Integrin signaling were deregulated as well as the PID Beta1, Beta2, and Beta3 Integrin Cell Surface Interactions pathways (Additional file 3: Table S4). As previously noted, the radiation treatment assignment cohort also exhibited gene expression aberrancies in extracellular matrix interactions indicating similar biology in this subgroup (Additional file 3: Table S5). This supports the concept that microenvironmental interactions involving integrins are essential for HNSCC progression.

### IL-10 signaling alterations in HNSCC progressors

Notably, the IL-10 Anti-inflammatory Signaling pathway was significantly enriched for putative differentially expressed genes among HNSCC progressors (FDR adjusted P-value 0.042, Fig. 2, Additional file 3: Table S4). In addition, HNSCC progressors also harbored unique mutations in several members of the pathway including *IL-6, STATs including STAT5A, and BLVRB* (Fig. 2). Specifically, 41.2 % of the progressors had at least one gene overexpressed in this pathway (with a maximum of 38.5 % of the pathway overexpressed observed in any progressor) and 11.8 % of the progressors had at least one gene mutated. This was intriguing given the early promise of immune-based therapies in HNSCC. In addition, multiple clotting pathways were found to be differentially expressed (FDR < 0.05) including the Fibrinolysis Pathway (Biocarta), the Intrinsic Prothrombin Activation Pathway (Biocarta), Genes involved in Platelet Aggregation (Reactome), and Complement and Coagulation Cascades (KEGG). These pathways are linked to inflammation [4] as well, and could also point to the importance of this microenvironmental alteration in HNSCC progression. Again, similar gene expression deregulation was seen in these pathways for the radiation treatment assignment cohort.

### Gene signature predicting survival

**Fig. 4:**
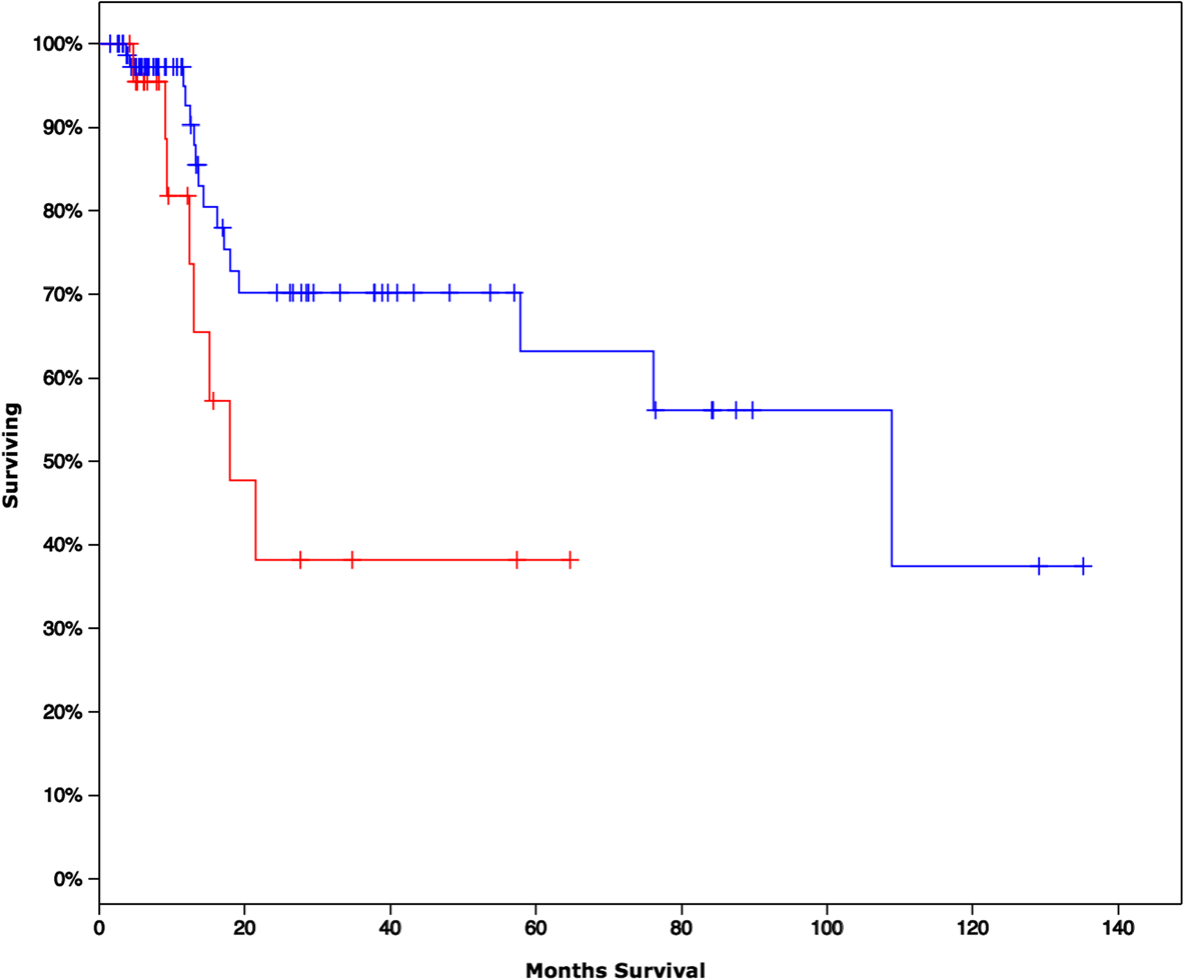
Overall Survival Kaplan-Maier estimates for gene signature (PAGE4, SMTNL1, VTN, CA5A, C1orf43, KRTAP19-1, LEP, HRH4, PAGE5, SEZ6L, CREB3; Logrank Test P-Value: 0. 03443). Censored patients are indicated by tick marks. Signature is based on those genes differentially expressed and also mutated only in progressors. Red indicates cases with alterations in those genes (based on Z > 2.5) Median survival in months for those with alterations in the gene signature is 17.94 months compared to 108.88 months for those without the alterations

Given the clear unique molecular alterations in HNSCC progressors, we were able to generate an 11-gene signature predicting survival based on those genes both differentially expressed and mutated only in progressors. Overall survival using Kaplan-Maier estimates for those patients harboring the gene signature (*PAGE4, SMTNL1, VTN, CA5A, C1orf43, KRTAP19-1, LEP, HRH4, PAGE5, SEZ6L, CREB3*) was significantly diminished (Logrank Test P-Value: 0. 03443). Median survival for those with alterations in the gene signature was 17.94 months compared to 108.88 months for those without the alterations (Fig. 4). Of note several of these genes are involved in interactions with the extracellular environment including *VTN* (vitronectin) and *KRTAP19-1* (a keratin associated protein).

### Cox modeling of molecular and clinical data

Given the prognostic ability of the gene signature above, we were interested in whether we could model survival based on these molecular changes and historically important clinical factors. We examined key clinical features (nodal extracapsular spread, alcohol use, tobacco smoking history, gender, margin status) as well as the combined mutation and expression gene signature using a multivariate Cox proportional hazards modeling approach. While the overall model was significant (*P* = 0.032), the key factors were interestingly nodal extracapsular spread and alcohol use (*P* = 0.004 for model with those factors alone).

## Conclusions

Mining the TCGA database provides unprecedented opportunities to unravel unique feature of tumorigenesis in HNSCC. Recent published analysis of primary HNSCC molecular alterations in patient data from TCGA reiterated known HNSCC drivers and uncovered distinct molecular alterations between HPV and tobacco-driven tumors [3]. Tumor heterogeneity regardless of the pathways involved was linked to reduced overall survival in a companion study of this population, highlighting the genomically unstable nature of this cancer [5]. The heterogeneity of HNSCC, in part, has limited improved therapeutic targeting of this disease. In fact, cetuximab, targeting EGFR overexpression, is the only targeted agent used in the treatment of HNSCC. However single agent response rates are low, and in combination with standard chemotherapy for progressive disease, overall survival remains less than one year [2]. Many groups are harnessing the power of the TCGA data to characterize molecular changes that might predict survival, exemplified by a recent study suggesting an 11-gene signature was able to predict nodal extracapsular spread and also overall survival [6]. Our study was designed to identify the genomic differences between progressors and nonprogressors at both the DNA and RNA level in order to highlight important pathways associated with progression. Interestingly, we uncovered a significant increase in deleterious mutations of *CTCF*, which is a master chromatin regulator associated with genomic instability and cancer progression [7, 8]. Deregulation of this gene could be a contributor to the genomic instability and heterogeneity in HNSCC although further mechanistic studies would be required for evaluation. Interestingly, progressors displayed differentially expressed genes harboring *CTCF* binding sites that participated Integrin-related pathways. This indicates that at least one potential downstream effect of *CTCF* deregulation could be aberrant microenvironmental interactions involving Integrins facilitating HNSCC progression.

We identifed Integrin and IL-10 signaling as unique prognostic pathways for HNSCC progression. Microenvironmental interaction aberrancies were confirmed by both mutational and expression analysis, and was revealed in progressor cohorts irrespective of their radiation treatment assignment. This implicates tumor microenvironment interactions in the driving biology of tumor progression for all HNSCC patients including those that require radiation as part of their treatment. Intriguingly, both of these pathways have potential promise for guiding targeting therapies. Recently, targeting both EGFR overexpression and Integrin B1 signaling was shown to radiosensitize HNSCC cells, building on previous literature demonstrating Integrin aberrations in HNSCC [9]. Further, cilengitide, an αvβ3 and αvβ5 Integrin inhibitor, has been tested in the recurrent/metastatic HNSCC setting in combination with cytotoxic chemotherapy, however there was no improvement in progression free survival with addition of cilengitide [10]. This should be reevaluated with the improved biomarkers identified in this study or in the definitive rather than metastatic setting. Alterations in IL-10 signaling uncovered in this study suggests to an interesting therapeutic angle. Inflammation is a hallmark of HNSCC progression based on both animal and human studies [11, 12]. IL-10 signaling plays a key role in regulation of cancer-associated inflammation including regulation of CD8 T cells [13]. As targeting of CD8 cells with PD1 (programmed death 1) pathway inhibitors has shown significant promise in multiple similar tumor types, it has emerged as a attractive targetable pathway in HNSCC [14]. Potentially, deregulation of the IL-10 pathway could be used as a biomarker to stratify patients more likely to respond to this therapy. Finally, we identified an 11-gene signature to predict for progression. In addition to alcohol use and nodal extracapsular spread, a known poor prognostic pathologic factor utilized by other groups [6], this pathway was very powerful in stratifying patients with poor prognosis. Our novel gene signature could be used to identify patients that could benefit from intensified therapy (either concurrent with definitive therapy or adjuvant). The limitations of our study include that we were unable to stratify by HPV status given incomplete clinical annotation within the TCGA dataset, we did not have access to recurrent tumor tissue, and we did not stratify by stage. Nevertheless we were able to uncover significant aberrant pathways that, after further mechanistic validation, have potential to open new avenues for therapeutic treatment of recurrent HNSCC.

## Methods

### Selection of patients and study design

TCGA HNSCC data used for this analysis were time stamped August 13^th^ 2014 and downloaded from the TCGA Research Network: http://cancergenome.nih.gov/. Data types utilized were the clinical data (patient demographics, drug therapy, radiation therapy, and follow-up), RNA-Seq V2 (Level 3; Illumina HiSeq 2000), and somatic mutations (Level 2; Illumina Genome Analyzer DNA Sequencing). All data was mapped to genome build hg 19.

Patients were first classified as progressor or nonprogressor based on follow-up annotation, specificaly the presence or absence of a new tumor event. We required annotation to confirm the tumor event (days to new tumor and/or new tumor anatomical location). All patients were required to have treatment annotation in addition to the follow-up data. All samples used in this study were collected from initial pre-treatment diagnosis (the samples had not been exposed to chemotherapy or radiation).

### Statistical analysis

In-house workflows in the R Statistical Programming environment were used for all QA/QC and analysis [15]. The clinical data was checked for duplicated rows, blank fields and other quality checks. All of the clinical data sets were merged together by the common BCR Patient Barcodes. Differential expression (DE) analysis between progressors and nonprogressors was conducted by fitting linear models using the edgeR framework [16]. As a secondary analysis, we also examined differential expression among progressors and nonprogressors with radiation treatment assignment. For all DE, P-values were False Discovery Rate Adjusted. Genes with low expression in all samples (<1 (count per million) were flagged and filtered out. Somatic mutation data was also filtered out if there were any tuples with no known gene symbols in RNA-Seq V2. All gene symbols were verified to have approved symbols or synonyms. Cytoscape was used for stylized pathway visualization [17] specifically the Reactome FI Cytoscape Plugin 4 [18].

Somatic mutations for progressors and nonprogressors were evaluated by first assessing gene symbol, chromosome, and start and stop. The distribution of truncating mutations (nonsense, nonstop, frameshift deletion, frameshift insertion, and splice site) as well as missense mutation rates was compared between progressors and nonprogressors. For the entire TCGA HNSCC cohort, MutSig2CV annotation (ranking and significance) was examined to assess unique mutations. MutSig analyzes mutations to identify genes that were mutated more often than expected by chance given background mutation processes [19]. Fisher’s exact tests were performed to examine differences in mutational frequency by mutational class in those genes with overall mutational frequency difference of 5 % or more between progressors and nonprogressors. Lollipop figures of mutational type by gene were generated by the MutationMapper visualization tool (courtesy of Memorial Sloan-Kettering Cancer Center). Somatic mutations were counted both as the number of total of variants, as well as summarized at the gene level as the total number of genes mutated. For mutations unique to progressors and unique to nonprogressors (noting the caveat this can in some cases be due to sampling), we computed the ratio of variants to gene level mutation (in both cases only for those mutations unique to each group) = # of somatic mutations (individual variants) / # of genes with somatic mutations. This allowed us to assess the number of mutations relative to the number of genes mutated to understand the impact on the genome. A large ratio could be indicative a concentration of highly mutated genes.

Both differentially expressed genes as well as somatic mutation data were annotated to pathway models from Reactome, KEGG, Pathway Interaction Database (PID), and Biocarta from MSIGDB [20]. We then examined if there was significant enrichment of these candidate genes in the pathways. As with the differential expression analysis, all enrichment *P*-values were False Discovery Rate Adjusted.

Mutation and gene expression data was overlaid to identify an aggregate gene signature (based on both differentially expression and unique mutation in progressors only). Overall Survival Kaplan-Maier estimates based on alternations in this signature were examined. Both clinical features (nodal extracapsular spread, alcohol use, tobacco smoking history, gender, margin status) as well as the aggregated gene signature were examined by a multivariate Cox proportional hazards modeling approach.

## Additional files

Additional file 1: Table S1. Demographics for TCGA HNSCC Progressors. Table S2. Demographics for TCGA HNSCC Non-progressors. (DOCX 484 kb) Additional file 2: Table S3. Putative Differential Expression (DE) between HNSCC TCGA Annotated Progressors and NonProgressors (False Discovery Rate (FDR) < 0.05). CTCF binding site annotation was from CTCFBSDB 2.0. (DOCX 489 kb) Additional file 3: Table S4. Pathways significantly enriched for differentially expressed (DE) genes between TCGA HNSCC progressors and nonprogressors. FDR = False Discovery Rate. Table S5. Pathways significantly enriched for differentially expressed (DE) genes between TCGA HNSCC progressors and nonprogressors who were assigned radiation treatment. FDR = False Discovery Rate. (DOCX 488 kb) Additional file 4: Table S6. TCGA HNSCC Somatic Mutations in Progressors and NonProgressors. Truncated mutations are defined as Nonsense, Nonstop, Frameshift deletion, Frameshift insertion, and Splice site mutations. Data was examined in 68 Progressors (PR) and 163 NonPogressors (NP). (DOCX 483 kb)
