# Supplementary material for: IL-10 and integrin signaling pathways are associated with head and neck cancer progression
Source: BMC Genomics. 2016 Jan 8;17:38. doi: 10.1186/s12864-015-2359-6 (PMC4706689; doi:10.1186/s12864-015-2359-6)
Supplement: Additional file 1: Table S1. — Demographics for TCGA HNSCC Progressors. Table S2. Demographics for TCGA HNSCC Non-progressors. (DOCX 484 kb) [file 12864_2015_2359_MOESM1_ESM.docx]

**Supplementary Tables 1-2**

**Table 1. Demographics for TCGA HNSCC Progressors**

|  | **Patients (*n*=68)** |
| --- | --- |
| **Age (mean)** | 39-88 (62) |
| **Gender (M/F)** | 52(76%)/16(24%) |
| **Race (W/B/A/AI/NA)*** | 62(91%)/4(6%)/1(1.5%)/0/1(1.5%) |
| **Smoke (Y/N/NA)** | 53(78%)/15(22%)/0 |
| **Alcohol (Y/N/NA)** | 53(78%)/13(19%)/2(3%) |
| **HPV p16 or ISH (+/-/NA)** | 2(3%)/14(21%)/52(76%) |
| **Site (OC/OPX/L)**** | 38(56%)/13(19%)/17(25%) |
| **T Stage (T1-T2/T3/T4/TX/NA)** | 25(36.5%)/19(28%)/23(34%)/1(1.5%)/0 |
| **N Stage (N0/N+/NA)** | 31(46%)/37(54%)/0 |
| **Tumor Stage (I-III/IV/NA)** | 25(36.5%)/42(62%)/1(1.5%) |
| **Margin Status (+/-/Close/NA)** | 14(20.5%)/39(57.5%)/6(9%)/9(13%) |
| **Nodal Extracapsular Spread (GE/ME/NE/NA)***** | 10(15%)/14(21%)/24(35%)/20(29%) |
| **Therapy (C/R/CR/CRTM/CRTMV/NA)****** | 3(4.5%)/18(26.5%)/39(57.5%)/0/1(1.5%)/7(10%) |
| **Radiation Dose cGy (mean)** | 42-7200 (5283) |
| **Follow-up Days (median)** | 46-4241 (411) |
| **Mortality (L/D)******* | 26(38%)/42(62%) |
| **Days to Death (median)** | 129-3314 (475.5) |
| **Days to New Tumor (median)** | 50-1859 (339) |
| **Radiation Treatment (Y/N)** | 60(88%)/8(12%) |
| *W=White; B=Black; A=Asian; AI=American Indian  **OC=Oral Cavity; OPX=Oropharynx; L=Larynx  ***GE=Gross Extension; ME=Microscopic Extension; NE=No Extranodal Extension  ****C=Chemotherapy Only; R=Radiation only; CR=Chemotherapy & Radiation; CRTM=Chemotherapy, Radiation & Targeted Molecular Therapy; CRTMV= Chemotherapy, Radiation, Targeted Molecular Therapy & Vaccine  *****L=Living; D=Deceased | |

**Table 2. Demographics for TCGA HNSCC Non-progressors**

|  | **Patients (*n*=167)** |
| --- | --- |
| **Age (mean)** | 20-90 (61.5) |
| **Gender (M/F)** | 121(72%)/46(28%) |
| **Race (W/B/A/AI/NA)*** | 150(90%)/8(5%)/3(2%)/2(1%)/4(2%) |
| **Smoke (Y/N/NA)** | 128(77%)/37(22%)/2(1%) |
| **Alcohol (Y/N/NA)** | 116(69%)/48(29%)/3(2%) |
| **HPV p16 or ISH (+/-/NA)** | 17(10%)/35(21%)/115(69%) |
| **Site (OC/OPX/L)**** | 97(58%)/28(17%)/42(25%) |
| **T Stage (T1-T2/T3/T4/TX/NA)** | 55(33%)/45(27%)/64(38.5%)/2(1%)/1(0.5%) |
| **N Stage (N0/N+/NA)** | 76(45.5%)/90(54%)/1(0.5%) |
| **Tumor Stage (I-III/IV/NA)** | 71(42.5%)/93(55.5%)/3(2%) |
| **Margin Status (+/-/Close/NA)** | 3(2%)/119(71%)/16(10%)/29(17%) |
| **Nodal Extracapsular Spread (GE/ME/NE/NA)***** | 3(2%)/20(12%)/90(54%)/54(32%) |
| **Therapy (C/R/CR/CRTM/CRTMV/NA)****** | 2(1%)/42(25%)/56(34%)/1(0.5%)/0/66(39.5%) |
| **Radiation Dose cGy (mean)** | 9-7380 (4405) |
| **Follow-up Days (median)** | 45-3930 (566.5) |
| **Mortality (L/D)******* | 138(83%)/29(17%) |
| **Days to Death (median)** | 23-5152 (397) |
| **Days to New Tumor (median)** | NA |
| **Radiation Treatment (Y/N)** | 105(63%)/62(37%) |
| *W=White; B=Black; A=Asian; AI=American Indian  **OC=Oral Cavity; OPX=Oropharynx; L=Larynx  ***GE=Gross Extension; ME=Microscopic Extension; NE=No Extranodal Extension  ****C=Chemotherapy Only; R=Radiation only; CR=Chemotherapy & Radiation; CRTM=Chemotherapy, Radiation & Targeted Molecular Therapy; CRTMV= Chemotherapy, Radiation, Targeted Molecular Therapy & Vaccine  *****L=Living; D=Deceased | |
