# Supplementary material for: IL-10 and integrin signaling pathways are associated with head and neck cancer progression
Source: BMC Genomics. 2016 Jan 8;17:38. doi: 10.1186/s12864-015-2359-6 (PMC4706689; doi:10.1186/s12864-015-2359-6)
Supplement: Additional file 2: Table S3. — Putative Differential Expression (DE) between HNSCC TCGA Annotated Progressors and NonProgressors (False Discovery Rate (FDR) < 0.05). CTCF binding site annotation was from CTCFBSDB 2.0. (DOCX 489 kb) [file 12864_2015_2359_MOESM2_ESM.docx]

**Supplemental Table 3. Putative Differential Expression (DE) between HNSCC TCGA Annotated Progressors and NonProgressors (False Discovery Rate (FDR) < 0.05). CTCF binding site annotation was from CTCFBSDB 2.0**

| **Gene Symbol** | **Entrez ID** | **logFC** | **P-Value** | **FDR** | **Mutated**  **Progressors**  **Only** | **CTCF**  **Binding**  **Site** |
| --- | --- | --- | --- | --- | --- | --- |
| ABT1 | 29777 | 0.385595 | 0.000033 | 0.013931 |  | YES |
| ACTL8 | 81569 | 1.979446 | 0.000149 | 0.036297 |  | YES |
| ALB | 213 | 2.205318 | 0.000041 | 0.015750 |  | YES |
| AQP4 | 361 | 1.900435 | 0.000027 | 0.012184 |  | YES |
| ARMC1 | 55156 | 0.411826 | 0.000080 | 0.024972 |  | YES |
| ASNSD1 | 54529 | 0.384263 | 0.000023 | 0.011217 |  |  |
| ATP2A1 | 487 | 1.787786 | 0.000185 | 0.042134 |  | YES |
| ATP2C1 | 27032 | 0.463469 | 0.000008 | 0.005261 |  |  |
| BIRC2 | 329 | 1.263228 | 0.000000 | 0.000008 |  |  |
| BPHL | 670 | 0.465703 | 0.000208 | 0.043560 |  |  |
| BTBD17 | 388419 | 2.142139 | 0.000122 | 0.032259 |  |  |
| C11orf59 | 55004 | 0.565623 | 0.000164 | 0.038790 |  |  |
| C11orf70 | 85016 | 1.383158 | 0.000038 | 0.014960 |  | YES |
| C15orf62 | 643338 | -1.172943 | 0.000153 | 0.036935 |  |  |
| C1GALT1 | 56913 | 0.549776 | 0.000038 | 0.014960 |  |  |
| C1orf43 | 25912 | 0.319941 | 0.000084 | 0.025826 | YES |  |
| C3orf43 | 255798 | 1.794923 | 0.000004 | 0.002931 |  |  |
| C7orf70 | 84792 | 0.425491 | 0.000033 | 0.013931 |  |  |
| CA5A | 763 | 2.313160 | 0.000001 | 0.000762 | YES | YES |
| CCDC107 | 203260 | 0.788220 | 0.000023 | 0.011217 |  |  |
| CKLF | 51192 | 0.573414 | 0.000019 | 0.011217 |  |  |
| CLC | 1178 | 2.069985 | 0.000023 | 0.011217 |  |  |
| CMBL | 134147 | 0.778233 | 0.000148 | 0.036297 |  |  |
| COMMD10 | 51397 | 0.471884 | 0.000054 | 0.018698 |  | YES |
| COMMD2 | 51122 | 0.410447 | 0.000099 | 0.028577 |  | YES |
| CREB3 | 10488 | 0.832730 | 0.000000 | 0.000095 | YES | YES |
| EPHA8 | 2046 | 1.622900 | 0.000228 | 0.046059 |  | YES |
| EPS8L1 | 54869 | -1.304613 | 0.000021 | 0.011217 |  |  |
| FAM49B | 51571 | 0.403064 | 0.000121 | 0.032259 |  |  |
| FAM86C | 55199 | 0.750075 | 0.000022 | 0.011217 |  |  |
| FASTKD3 | 79072 | 0.494057 | 0.000197 | 0.042995 |  | YES |
| FBXO4 | 26272 | 0.582144 | 0.000124 | 0.032259 |  |  |
| FGA | 2243 | 2.366307 | 0.000004 | 0.003489 |  |  |
| FGB | 2244 | 3.029749 | 0.000000 | 0.000196 |  | YES |
| FGG | 2266 | 2.675606 | 0.000000 | 0.000214 |  | YES |
| FLOT1 | 10211 | 0.398273 | 0.000098 | 0.028577 |  |  |
| FSIP1 | 161835 | 0.963154 | 0.000067 | 0.022013 |  | YES |
| FZD6 | 8323 | 0.538997 | 0.000050 | 0.017931 |  | YES |
| GALR2 | 8811 | 2.022381 | 0.000000 | 0.000000 |  | YES |
| GBA2 | 57704 | 0.626902 | 0.000027 | 0.012184 |  |  |
| GBAS | 2631 | 0.772894 | 0.000021 | 0.011217 |  |  |
| GC | 2638 | 3.015240 | 0.000010 | 0.006210 |  | YES |
| GLO1 | 2739 | 0.518266 | 0.000065 | 0.021996 |  |  |
| GRIK5 | 2901 | -1.622485 | 0.000224 | 0.045922 |  | YES |
| GYG1 | 2992 | 0.523927 | 0.000165 | 0.038790 |  |  |
| HAX1 | 10456 | 0.329624 | 0.000205 | 0.043399 |  |  |
| HIBADH | 11112 | 0.463372 | 0.000023 | 0.011217 |  | YES |
| HMOX1 | 3162 | 1.077314 | 0.000000 | 0.000416 |  |  |
| HRH4 | 59340 | 2.087266 | 0.000000 | 0.000000 | YES | YES |
| HTR3C | 170572 | 2.318397 | 0.000000 | 0.000004 |  |  |
| IL8 | 3576 | 1.318387 | 0.000129 | 0.032732 |  | YES |
| KIAA1377 | 57562 | 1.327713 | 0.000004 | 0.002931 |  | YES |
| KIAA1430 | 57587 | 0.449510 | 0.000190 | 0.042912 |  |  |
| KLHL38 | 340359 | 1.460837 | 0.000068 | 0.022013 |  |  |
| KRT36 | 8689 | -3.908459 | 0.000167 | 0.038852 |  |  |
| KRTAP19-1 | 337882 | 2.659232 | 0.000001 | 0.000983 | YES |  |
| LEP | 3952 | 2.251866 | 0.000003 | 0.002438 | YES |  |
| LHX3 | 8022 | 1.864578 | 0.000051 | 0.017931 |  | YES |
| LOC121952 | 121952 | 1.784680 | 0.000110 | 0.030837 |  |  |
| LRTOMT | 220074 | 0.560763 | 0.000218 | 0.045111 |  |  |
| MGC14436 | 84983 | 1.451284 | 0.000071 | 0.022597 |  | YES |
| MRS2 | 57380 | 0.412599 | 0.000073 | 0.022997 |  |  |
| NCRNA00110 | 642976 | 2.659985 | 0.000000 | 0.000003 |  |  |
| NKPD1 | 284353 | -1.377882 | 0.000246 | 0.048015 |  |  |
| NSMCE2 | 286053 | 0.471163 | 0.000037 | 0.014960 |  | YES |
| OLIG1 | 116448 | 2.368841 | 0.000001 | 0.000983 |  |  |
| PAGE4 | 9506 | 2.366931 | 0.000026 | 0.012184 | YES | YES |
| PAGE5 | 90737 | -4.501146 | 0.000198 | 0.042995 | YES | YES |
| PCDHGB8P | 56120 | 2.005946 | 0.000000 | 0.000000 |  |  |
| PEX2 | 5828 | 0.463024 | 0.000032 | 0.013931 |  |  |
| PKD1L2 | 114780 | 1.366942 | 0.000143 | 0.035785 |  |  |
| PLCXD1 | 55344 | -0.866500 | 0.000127 | 0.032599 |  |  |
| PPIL1 | 51645 | 0.562195 | 0.000001 | 0.000983 |  | YES |
| PPP2R3C | 55012 | 0.368635 | 0.000119 | 0.032259 |  | YES |
| PRDM16 | 63976 | 1.006428 | 0.000124 | 0.032259 |  | YES |
| PSG9 | 5678 | 1.848565 | 0.000006 | 0.004224 |  |  |
| PSPH | 5723 | 0.657978 | 0.000199 | 0.042995 |  |  |
| PYGM | 5837 | 1.811215 | 0.000177 | 0.040663 |  |  |
| RPS18 | 6222 | 0.546060 | 0.000204 | 0.043399 |  |  |
| SERHL2 | 253190 | 0.902094 | 0.000005 | 0.003816 |  |  |
| SEZ6L | 23544 | 1.924286 | 0.000005 | 0.003538 | YES | YES |
| SLC26A3 | 1811 | 2.852290 | 0.000002 | 0.001890 |  | YES |
| SLC38A6 | 145389 | 0.587110 | 0.000009 | 0.005669 |  | YES |
| SMTNL1 | 219537 | 1.554309 | 0.000231 | 0.046059 | YES |  |
| SOX14 | 8403 | 3.305343 | 0.000044 | 0.016540 |  | YES |
| SPANXC | 64663 | 2.639341 | 0.000041 | 0.015750 |  |  |
| SPINK6 | 404203 | -3.561626 | 0.000199 | 0.042995 |  | YES |
| SPINK7 | 84651 | -4.002204 | 0.000046 | 0.016806 |  |  |
| STAT4 | 6775 | 2.067460 | 0.000000 | 0.000000 |  | YES |
| STK17A | 9263 | 0.657511 | 0.000029 | 0.012939 |  |  |
| SUV420H1 | 51111 | 0.462531 | 0.000095 | 0.028307 |  |  |
| TCTEX1D1 | 200132 | 1.311024 | 0.000000 | 0.000000 |  | YES |
| TESK1 | 7016 | 1.018138 | 0.000000 | 0.000000 |  | YES |
| TIPRL | 261726 | 0.336920 | 0.000102 | 0.029079 |  |  |
| TMEM14B | 81853 | 0.584474 | 0.000003 | 0.002438 |  |  |
| TMEM14C | 51522 | 0.583215 | 0.000000 | 0.000642 |  |  |
| TMEM194B | 100131211 | 0.684800 | 0.000005 | 0.003816 |  |  |
| TMEM88 | 92162 | 0.801343 | 0.000123 | 0.032259 |  |  |
| TPCN2 | 219931 | 0.875510 | 0.000012 | 0.007206 |  |  |
| TUBA4B | 80086 | 1.672430 | 0.000065 | 0.021996 |  |  |
| UGT2B11 | 10720 | 2.938766 | 0.000000 | 0.000134 |  |  |
| VTN | 7448 | 1.443490 | 0.000002 | 0.001597 | YES | YES |
| WFDC12 | 128488 | -3.085781 | 0.000236 | 0.046616 |  |  |
| ZFAND1 | 79752 | 0.375099 | 0.000231 | 0.046059 |  |  |
| ZNF410 | 57862 | 0.401367 | 0.000095 | 0.028307 |  |  |
