# Supplementary material for: IL-10 and integrin signaling pathways are associated with head and neck cancer progression
Source: BMC Genomics. 2016 Jan 8;17:38. doi: 10.1186/s12864-015-2359-6 (PMC4706689; doi:10.1186/s12864-015-2359-6)
Supplement: Additional file 3: Table S4. — Pathways significantly enriched for differentially expressed (DE) genes between TCGA HNSCC progressors and nonprogressors. FDR = False Discovery Rate. Table S5. Pathways significantly enriched for differentially expressed (DE) genes between TCGA HNSCC progressors and nonprogressors who were assigned radiation treatment. FDR = False Discovery Rate. (DOCX 488 kb) [file 12864_2015_2359_MOESM3_ESM.docx]

**Supplemental Table 4. Pathways significantly enriched for differentially expressed (DE) genes between TCGA HNSCC progressors and nonprogressors. FDR = False Discovery Rate**

| **Source** | **Pathway Size** | **Description** | **#DE Gene Members** | **P-value** | **FDR-adjusted P-value** |
| --- | --- | --- | --- | --- | --- |
| Biocarta | 12 | Fibrinolysis Pathway | 3 | 2.37E-06 | 3.34E-04 |
| PID | 42 | Urokinase-type plasminogen activator (uPA) and uPAR-mediated signaling | 4 | 2.49E-06 | 9.20E-04 |
| PID | 43 | Beta3 integrin cell surface interactions | 4 | 2.75E-06 | 9.20E-04 |
| Biocarta | 13 | Extrinsic Prothrombin Activation Pathway | 3 | 3.08E-06 | 3.34E-04 |
| Reactome | 14 | Genes involved in Common Pathway | 3 | 3.91E-06 | 1.10E-03 |
| Reactome | 15 | Genes involved in GRB2:SOS provides linkage to MAPK signaling for Intergrins | 3 | 4.88E-06 | 1.10E-03 |
| Reactome | 15 | Genes involved in p130Cas linkage to MAPK signaling for integrins | 3 | 4.88E-06 | 1.10E-03 |
| Biocarta | 20 | Acute Myocardial Infarction | 3 | 1.21E-05 | 8.77E-04 |
| PID | 66 | Beta1 integrin cell surface interactions | 4 | 1.54E-05 | 2.26E-03 |
| Biocarta | 23 | Intrinsic Prothrombin Activation Pathway | 3 | 1.87E-05 | 1.02E-03 |
| Reactome | 27 | Genes involved in Integrin alphaIIb beta3 signaling | 3 | 3.08E-05 | 4.24E-03 |
| Reactome | 79 | Genes involved in Integrin cell surface interactions | 4 | 3.14E-05 | 4.24E-03 |
| PID | 29 | Beta2 integrin cell surface interactions | 3 | 3.83E-05 | 3.74E-03 |
| PID | 30 | Ephrin B reverse signaling | 3 | 4.25E-05 | 3.74E-03 |
| Reactome | 413 | Genes involved in Transmembrane transport of small molecules | 7 | 4.25E-05 | 4.36E-03 |
| Reactome | 89 | Genes involved in Response to elevated platelet cytosolic Ca2+ | 4 | 5.02E-05 | 4.36E-03 |
| Reactome | 32 | Genes involved in Formation of Fibrin Clot (Clotting Cascade) | 3 | 5.17E-05 | 4.36E-03 |
| Reactome | 36 | Genes involved in Platelet Aggregation (Plug Formation) | 3 | 7.40E-05 | 5.54E-03 |
| Reactome | 55 | Genes involved in Ion channel transport | 3 | 2.64E-04 | 1.78E-02 |
| KEGG | 69 | Complement and coagulation cascades | 3 | 5.14E-04 | 3.40E-02 |
| Biocarta | 17 | IL-10 Anti-inflammatory Signaling Pathway | 2 | 6.62E-04 | 2.87E-02 |
| Reactome | 18 | Genes involved in Glycogen breakdown (glycogenolysis) | 2 | 7.43E-04 | 4.56E-02 |
| PID | 82 | Glucocorticoid receptor regulatory network | 3 | 8.51E-04 | 4.89E-02 |

**Supplemental Table 5. Pathways significantly enriched for differentially expressed (DE) genes between TCGA HNSCC progressors and nonprogressors who were assigned radiation treatment. FDR = False Discovery Rate**

| **Source** | **Pathway Size** | **Description** | **#DE Gene Members** | **P-value** | **FDR-adjusted P-value** |
| --- | --- | --- | --- | --- | --- |
| Reactome | 279 | Genes involved in Neuronal System | 14 | 1.19E-06 | 7.91E-04 |
| Reactome | 396 | Genes involved in Developmental Biology | 16 | 3.52E-06 | 7.91E-04 |
| Reactome | 22 | Genes involved in FGFR ligand binding and activation | 5 | 2.35E-06 | 7.91E-04 |
| Reactome | 28 | Genes involved in SHC-mediated cascade | 5 | 8.33E-06 | 1.40E-03 |
| Reactome | 251 | Genes involved in Axon guidance | 12 | 1.11E-05 | 1.46E-03 |
| Reactome | 139 | Genes involved in Biological oxidations | 9 | 1.30E-05 | 1.46E-03 |
| KEGG | 72 | Drug metabolism - cytochrome P450 | 7 | 8.55E-06 | 1.53E-03 |
| KEGG | 272 | Neuroactive ligand-receptor interaction | 12 | 2.46E-05 | 1.53E-03 |
| KEGG | 55 | Steroid hormone biosynthesis | 6 | 1.96E-05 | 1.53E-03 |
| Reactome | 408 | Genes involved in GPCR ligand binding | 15 | 2.14E-05 | 2.06E-03 |
| Reactome | 36 | Genes involved in FRS2-mediated cascade | 5 | 2.99E-05 | 2.10E-03 |
| Reactome | 121 | Genes involved in G alpha (s) signalling events | 8 | 3.38E-05 | 2.10E-03 |
| Reactome | 35 | Genes involved in Metabolism of steroid hormones and vitamins A and D | 5 | 2.60E-05 | 2.10E-03 |
| Reactome | 37 | Genes involved in Negative regulation of FGFR signaling | 5 | 3.43E-05 | 2.10E-03 |
| KEGG | 129 | Axon guidance | 8 | 5.34E-05 | 2.48E-03 |
| Reactome | 98 | Genes involved in Potassium Channels | 7 | 6.41E-05 | 3.60E-03 |
| KEGG | 80 | Cardiac muscle contraction | 6 | 1.64E-04 | 5.37E-03 |
| KEGG | 28 | Pentose and glucuronate interconversions | 4 | 1.73E-04 | 5.37E-03 |
| Reactome | 78 | Genes involved in Platelet homeostasis | 6 | 1.43E-04 | 7.39E-03 |
| Reactome | 12 | Genes involved in FGFR4 ligand binding and activation | 3 | 2.10E-04 | 8.57E-03 |
| Reactome | 52 | Genes involved in Heparan sulfate/heparin (HS-GAG) metabolism | 5 | 1.81E-04 | 8.57E-03 |
| Reactome | 54 | Genes involved in Phospholipase C-mediated cascade | 5 | 2.16E-04 | 8.57E-03 |
| Reactome | 29 | Genes involved in Steroid hormones | 4 | 1.99E-04 | 8.57E-03 |
| KEGG | 169 | Alzheimer's disease | 8 | 3.45E-04 | 8.92E-03 |
| KEGG | 267 | MAPK signaling pathway | 10 | 4.29E-04 | 8.92E-03 |
| KEGG | 135 | Oxidative phosphorylation | 7 | 4.67E-04 | 8.92E-03 |
| KEGG | 64 | Retinol metabolism | 5 | 4.80E-04 | 8.92E-03 |
| Reactome | 86 | Genes involved in L1CAM interactions | 6 | 2.44E-04 | 9.11E-03 |
| Reactome | 56 | Genes involved in PI-3K cascade | 5 | 2.57E-04 | 9.11E-03 |
| KEGG | 69 | Complement and coagulation cascades | 5 | 6.79E-04 | 1.03E-02 |
| KEGG | 71 | Melanoma | 5 | 7.73E-04 | 1.03E-02 |
| KEGG | 70 | Metabolism of xenobiotics by cytochrome P450 | 5 | 7.25E-04 | 1.03E-02 |
| KEGG | 69 | PPAR signaling pathway | 5 | 6.79E-04 | 1.03E-02 |
| Reactome | 305 | Genes involved in Class A/1 (Rhodopsin-like receptors) | 11 | 3.10E-04 | 1.04E-02 |
| Reactome | 14 | Genes involved in FGFR1 ligand binding and activation | 3 | 3.43E-04 | 1.05E-02 |
| Reactome | 466 | Genes involved in Hemostasis | 14 | 3.26E-04 | 1.05E-02 |
| Reactome | 15 | Genes involved in Unblocking of NMDA receptor, glutamate binding and activation | 3 | 4.25E-04 | 1.25E-02 |
| Reactome | 16 | Genes involved in Activated point mutants of FGFR2 | 3 | 5.19E-04 | 1.40E-02 |
| Reactome | 137 | Genes involved in Neurotransmitter Receptor Binding And Downstream Transmission In The Postsynaptic Cell | 7 | 5.10E-04 | 1.40E-02 |
| Reactome | 188 | Genes involved in Peptide ligand-binding receptors | 8 | 6.97E-04 | 1.67E-02 |
| Reactome | 70 | Genes involved in Phase II conjugation | 5 | 7.25E-04 | 1.67E-02 |
| Reactome | 70 | Genes involved in Phase 1 - Functionalization of compounds | 5 | 7.25E-04 | 1.67E-02 |
| Reactome | 18 | Genes involved in Platelet calcium homeostasis | 3 | 7.45E-04 | 1.67E-02 |
| Reactome | 186 | Genes involved in Transmission across Chemical Synapses | 8 | 6.50E-04 | 1.67E-02 |
| Reactome | 71 | Genes involved in PI3K Cascade | 5 | 7.73E-04 | 1.68E-02 |
| Biocarta | 15 | Nuclear Receptors in Lipid Metabolism and Toxicity | 3 | 4.25E-04 | 1.77E-02 |
| Reactome | 43 | Genes involved in Voltage gated Potassium channels | 4 | 9.27E-04 | 1.95E-02 |
| KEGG | 267 | Cytokine-cytokine receptor interaction | 9 | 1.70E-03 | 2.11E-02 |
| KEGG | 25 | Ascorbate and aldarate metabolism | 3 | 1.99E-03 | 2.32E-02 |
| KEGG | 134 | Cell adhesion molecules (CAMs) | 6 | 2.49E-03 | 2.57E-02 |
| KEGG | 133 | Parkinson's disease | 6 | 2.39E-03 | 2.57E-02 |
| Reactome | 49 | Genes involved in Nuclear Receptor transcription pathway | 4 | 1.52E-03 | 3.10E-02 |
| Reactome | 805 | Genes involved in GPCR downstream signaling | 18 | 1.60E-03 | 3.18E-02 |
| Reactome | 24 | Genes involved in Termination of O-glycan biosynthesis | 3 | 1.77E-03 | 3.40E-02 |
| Reactome | 87 | Genes involved in Insulin receptor signalling cascade | 5 | 1.92E-03 | 3.60E-02 |
| PID | 84 | C-MYB transcription factor network | 5 | 1.65E-03 | 4.35E-02 |
| PID | 24 | Visual signal transduction: Rods | 3 | 1.77E-03 | 4.40E-02 |
| Reactome | 56 | Genes involved in Cell-cell junction organization | 4 | 2.49E-03 | 4.53E-02 |
