# Supplementary material for: IL-10 and integrin signaling pathways are associated with head and neck cancer progression
Source: BMC Genomics. 2016 Jan 8;17:38. doi: 10.1186/s12864-015-2359-6 (PMC4706689; doi:10.1186/s12864-015-2359-6)
Supplement: Additional file 4: Table S6. — TCGA HNSCC Somatic Mutations in Progressors and NonProgressors. Truncated mutations are defined as Nonsense, Nonstop, Frameshift deletion, Frameshift insertion, and Splice site mutations. Data was examined in 68 Progressors (PR) and 163 NonPogressors (NP). (DOCX 483 kb) [file 12864_2015_2359_MOESM4_ESM.docx]

**Supplemental Table 6. TCGA HNSCC Somatic Mutations in Progressors and NonProgressors.**  Truncated mutations are defined as Nonsense, Nonstop, Frameshift deletion, Frameshift insertion, and Splice site mutations. Data was examined in 68 Progressors (PR) and 163 NonPogressors (NP).

| **All Mutations** | Progressors (PR) | NonProgressors (NP) | Intersection | Unique PR | Unique  NP |
| --- | --- | --- | --- | --- | --- |
| Number of Mutations (Variant Level) | 12557 | 36249 | 257 | 12300 | 35992 |
| Number of Mutations (Gene Level) | 7473 | 13144 | 5929 | 1544 | 7215 |
| **Variants/Gene Ratio** |  |  |  | **7.97** | **4.99** |
| **Range (Median) Gene Level** |  |  |  | 5-133 (21) | 3-845  (58) |

| **Truncated** | Progressors (PR) | NonProgressors (NP) | Intersection | Unique PR | Unique NP |
| --- | --- | --- | --- | --- | --- |
| Number of Mutations (Variant Level) | 1469 | 3959 | 41 | 1428 | 3918 |
| Number of Mutations (Gene Level) | 1266 | 2994 | 416 | 850 | 2578 |
| **Variants/Gene Ratio** |  |  |  | **1.68** | **1.52** |
